# Enriching table eggs with n-3 polyunsaturated fatty acids through dietary supplementation with the phototrophically grown green algae *Nannochloropsis limnetica*: effects of microalgae on nutrient retention, performance, egg characteristics and health parameters

**DOI:** 10.1016/j.psj.2022.101869

**Published:** 2022-03-24

**Authors:** A.J.W. Mens, M.M. van Krimpen, S.K. Kar, F.J. Guiscafre, L. Sijtsma

**Affiliations:** ⁎ Wageningen Livestock Research, Wageningen University & Research, P.O. Box 338, 6700 AH, Wageningen, the Netherlands; † Universidad Autónoma de Querétaro, Cerro de las campanas s/n, Santiago de Querétaro, C.P. 76010, Mexico; ‡ Wageningen Food & Biobased Research, Wageningen University & Research, 6700 AH, Wageningen, the Netherlands

**Keywords:** laying hens, microalgae, nutrition, eggs, omega-3

## Abstract

The fatty acid content of microalgae, especially the high content of omega-3 fatty acids such as eicosapentaenoic acid (**EPA**, C20:5) and docosahexaenoic acid (**DHA**, C22:6), could enrich eggs when fed to laying hens. Moreover, the properties and bioactive components of omega-3 fatty acids could positively influence the health and production performance of laying hens. In this study, the effects of dried *Nannochloropsis limnetica* inclusions in diets on yolk omega-3 fatty acid content, laying hen performance, nutrient retention, intestinal morphometry and systemic inflammatory markers were measured. A total of 240 twenty-five-wk-old laying hens were randomly assigned to 5 treatments distributed among 30 pens. Treatment A received the reference diet, while diets in treatments B, C, and D contained the control diet with 1, 2, and 3% microalgae added, respectively. In treatment E, a portion of ingredients of the control diet was replaced with rapeseed meal to induce a mild nutritional challenge, along with an inclusion of 3% microalgae. Compared to the control group the rate of lay increased by approximately 5% (*P* = 0.039) when birds were fed 2 or 3% microalgae. Furthermore, inclusion of 2 and 3% microalgae resulted in higher feed intake compared to the control group (126, 125, and 119 g/hen/d respectively; *P* = 0.001). Other performance parameters such as nutrient retention and egg characteristics were not affected by the dietary treatments. The EPA and DHA content of the yolk increased with increasing microalgae inclusion level (*P* < 0.001). A 2% algal inclusion resulted in 58.3 (**EPA**) and 603 (**DHA**) mg per 100 g dry yolk, respectively. Plasma haptoglobin levels of laying hens in both treatments receiving 3% microalgae were almost 3 times lower compared to the control group (1.25 and 1.62 vs. 5.60; *P* < 0.001), regardless of the inclusion of rapeseed in the diet. Based on these results, it can be concluded that the inclusion of *N. limnetica* enriches the egg yolk without negatively affecting the performance of laying hens and egg characteristics. Due to the positive effect on feed intake, microalgae in the diet provide nutritional benefits for laying hens. However, the positive effects of microalgae, especially on the health of laying hens, warrants further research.

## INTRODUCTION

Microalgae could be a more circular, sustainable, and promising feed ingredient for poultry diets. These aquatic unicellular organisms can potentially replace part of the protein sources, such as soya, used in most poultry diets (Świątkiewicz, et al. 2015; Madeira et al., 2017). Microalgae use CO_2_, nitrogen and phosphorus as main components and can grow on nutrient rich side streams such as for example, discharge water of some greenhouses. In such an approach, microalgae can clean the water by reducing the amounts of nitrogen and phosphorus and simultaneous produce a valuable product (Salazar et al., 2021). By selecting relevant strains, microalgal biomass might also compete in poultry diets with oils that are currently used as fat sources (i.e., palm oil, fish oil, or soya oil) or add specific functionalities (Świątkiewicz, et al. 2015; Madeira et al., 2017). Specific photosynthetic microalgae, containing high amounts of docosahexaenoic acid (**DHA**, C22:6 n-3) and eicosapentaenoic acid (**EPA**, C20:5 n-3), could be an interesting feedstuff for poultry diets due to the beneficial properties of omega-3 fatty acids on health and performance. Enriched eggs could help to increase omega-3 consumption in humans. From an animal nutrition point of view, it is important to know the extent to which the microalgae products can deliver digestible nutrients and to know the impact on production performance. In addition, it is relevant to determine the bioactive components in the algae products that can have health-promoting effects for the laying hens themselves. Microalgae contain natural pigments and include antibacterial, anti-inflammation, and antioxidant properties which have proven positive effects on gut health and immunity of humans and animals (Christaki et al., 2011; de Jesus Raposo et al., 2013; Noda et al., 2016; Furbeyre et al., 2017). The omega-3 fatty acids as well as some bioactive components in the microalgae products, like beta-glucans, might have health-promoting effects for the laying hens, as has been found in broiler chickens (Kang et al., 2013). This could improve the immune status of the hens, making them more resilient against infectious diseases. Regarding the poultry products (meat and eggs), microalgae in the diet of poultry could alter the fatty acid composition of the product. For example, by including microalgae in layer hen diets, the fatty acid composition of the yolk was altered due to the omega-3 fatty acids of the microalgae (Ginzberg et al., 2000; Fredriksson et al., 2006; Rizzi et al., 2009; Neijat et al., 2016). An increase in omega-3 fatty acids in the yolk, leads to more healthy and balanced products to use in human diets. Consequently, this might decrease costs related to healthcare of chronic diseases such as cardiovascular disease. Benefits for human health are mostly related to EPA and DHA, which are converted from alpha-linolenic acid (**ALA**, C18:3 n-3) (Trautwein, 2001). In humans, however, this conversion is very limited, making direct ingestion of omega-3 (with a focus on DHA and EPA) enriched food essential (Wu et al., 2019).

Results of an in vitro study conducted at our lab showed positive effect of microalgae on intestinal cells line (Hulst et al., submitted publication). Briefly, intestinal cells lines were incubated with microalgae and their potential effects were accessed by studying genome-wide transcriptome response. It emerged that microalgae positively affect numerous metabolic and immunological processes. However, there are many microalgae species available, and the exact properties and functions of each individual microalgae species needs to be established before it can be (safely) used in a poultry diet. Each individual microalgae species needs to be evaluated, both for functionality and nutritional values. However, the (bio)chemical composition of algae is not only species-specific but also depends on different environmental conditions (Kirpenko et al, 2015). Therefore, for commercial application of algal biomass in chicken feed, both the requirements of the nutritionists and the legal requirements must be met. The differences in algal composition and functional properties result in differences in digestibility and enrichment of the egg (Lemahieu et al., 2013). Furthermore, the optimal dosage requirement for microalgae to reach desired results for use as ‘functional’ feed-ingredients in laying hen diets under commercial setting and is technically viable and economically feasible, is not known.

This study focused on 2 aims: 1) the effects of dose dependent levels of the EPA producing freshwater microalgae *Nannochloropsis limnetica* in the diet of laying hens on nutrient retention, production performance and egg composition (fatty acid composition in the yolk), and 2) the effects of dietary *Nannochloropsis limnetica* on blood plasma parameters (cytokines and chemokines) and gut tissue by morphometry in the digestive tract.

## MATERIAL AND METHODS

This study was conducted from August until September 2019 at the animal research facilities of Wageningen University & Research (Lelystad, The Netherlands), in accordance with EU directive 2010/63 and approved by the Dutch Central Committee of Animal Experiments (The Hague, The Netherlands; protocol number: AVD401002015196).

### Housing and Management Laying Hens

In total 240 H&N Super Nick (Agromix, Lunteren, The Netherlands) laying hens (25 wk of age; 1,165.9 ± 29.3 g body weight) were purchased from a commercial laying hen farm. Hens were randomly allotted to 30 pens, with a minimum group weight difference of 5% (8 birds per pen). The pens were located in 2 identical, mechanically ventilated rooms and each room contained 15 pens (1.0 × 0.75 m) with flexible plastic slats. The flooring of the pens was covered with wood shavings and each pen contained a perch (0.75 m) and laying nest inside the pen. Feed was provided ad libitum in a trough adjacent to the pen and water was provided ad libitum by 2 drinking nipples per pen. The photoperiod consisted of 16 h light (04.00 to 20.00 h) with an illumination of 20 lux. During the experiment the temperature was kept at 21°C.

### Algal Biomass

Cells of the freshwater algae *Nannochloropsis limnetica* (CCMP2260, obtained from Bigelow laboratory, ME) were grown in well controlled horizontal photobioreactors, in filtered and UV treated discharge water (fresh water) from a greenhouse near Queretato, Mexico. Cells were harvested by centrifugation and oven-dried at 55 to 60°C before vacuum packaging and inclusion in the feed. The algal biomass was analyzed for nutrient content, pesticides, and microbial safety.

### Experimental Design and Experimental Diets

This study was performed using a completely randomized block design with 5 dietary treatments, three blocks per room and pen as experimental unit. Pens were randomly allotted to one of 5 dietary treatments within one block, each having 6 replicates. Dietary treatments consisted of three increasing inclusions (1, 2, and 3%) of the microalgae *N. limnetica,* a control group without the microalgae (reference diet) and a nutritional challenging treatment with 3% microalgae and part of the soybean meal and sunflower meal replaced by rapeseed meal. To obtain the 5 dietary treatments, 3 experimental diets were produced (Research Diet Service, Wijk bij Duurstede, the Netherlands):

1. CD: Control diet
2. CD3: Diet with 3% *N. limnetica*
3. RS3: Diet with 3% *N. limnetica* and 53% of soybean meal and 43% sunflower meal were replaced by rapeseed meal to induce a mild nutritional challenge.

Table 1 shows an overview of the treatments and the ratio of the 2 diets to obtain the dietary treatments. Experimental dietary treatment A was obtained from solely diet CD, experimental treatment D from solely CD3 and experimental dietary treatment E from solely RS3. The diets for experimental dietary treatments B and C were obtained by mixing 2/3 of diet CD and 1/3 of diet CD3, and 1/3 of diet CD and 2/3 of diet CD3, respectively. The experimental diets were isocaloric and isonitrogenous and based on commercial guidelines (CVB, 2018). The basal diet CD consisted of maize, wheat, sunflower meal, soybean meal, and palm oil as the main ingredients (Table 2). In the other experimental diets to include the microalgae, the CD3 diet was corrected for soybean meal, palm oil and monocalcium phosphate and the in the RS3 diet higher proportions of soybean meal and sunflower meal were replaced by the rapeseed meal, which additionally influenced the proportion of wheat, palm oil, and sunflower meal. All the diets were provided as a mash and were fed during the complete experimental period of 28 d.

**Table 1:** Overview of experimental treatments with increasing inclusion of microalgae *N. limnetica* and the ratio of experimental diets used to obtain the experimental treatments.

Diet	Algae inclusion (%)	RSM inclusion^1^	CD^2^ inclusion (%)	CD3^3^ inclusion (%)	RS3^4^ inclusion (%)
A	0	N	100	0	0
B	1	N	66.7	33.3	0
C	2	N	33.3	67.7	0
D	3	N	0	100	0
E	3	Y	0	0	100

1 Rapeseed meal exchanged part of the soybean meal and sunflower meal to induce a nutritional challenge.

2 CD = control diet.

3 CD3 = control diet with 3% *N. limnetica* inclusion.

4 RS3 = diet with 3% *N. limnetica* inclusion and 53% of soybean meal and 43% of sunflower meal replaced by rapeseed meal.

**Table 2:** Dietary ingredients and calculated nutrients of the experimental diets (g/kg, as-fed basis)

Diet^1^	CD	CD3	RS3
Ingredient			
Maize	400.0	400.0	400.0
Wheat	205.5	204.7	151.0
Sunflower meal	125.0	125.0	71.0
Soybean meal	115.2	96.2	45.4
Limestone	73.0	73.4	71.8
Palm oil	36.1	27.3	38.8
Chalk	20.0	20.0	20.0
Monocalcium phosphate	5.0	4.2	3.3
Premix^2^	5.0	5.0	5.0
Titanium dioxide	5.0	5.0	5.0
Salt	2.6	2.0	2.2
Phytase 1	2.0	2.0	2.0
L-Lysine HCl	2.0	2.1	1.8
Natrium-Bicarbonate	1.5	0.9	0.6
DL-Methionine	1.4	1.5	1.2
Phytase 2	0.5	0.5	0.5
L-Threonine	0.2	0.2	0.0
Rapeseed meal	0.0	0.0	150.0
L-Isoleucine	0.0	0.1	0.4
*Nannochloropsis Limnetica*	0.0	30.0	30.0
Calculated content^3^			
AME_n_ (MJ/kg)	11.5	11.5	11.5
DM	891.0	891.4	891.7
Crude ash	127.9	127.3	127.0
Crude protein	160.9	161.3	161.0
Crude fat	58.4	56.1	68.8
Crude fiber	36.8	36.5	43.4
Starch	379.2	378.7	354.4
Sugar	29.7	29.1	32.8
NDF	113.2	111.5	128.5
NSP	153.4	157.3	171.5
Dig. Lys	6.90	6.90	6.90
Dig. Met+Cys	6.10	6.10	6.10
Dig. Thr	4.80	4.80	4.80
Dig. Trp	1.50	1.50	1.50
Na	1.5	1.5	1.5
K	6.7	6.6	6.3
Cl	2.5	2.5	2.5
DEB (mEq/kg)	167.5	164.1	156.5
Ca	38.0	38.0	38.0
Total phosphorus	4.9	4.9	5.3
Available phosphorus	2.8	2.8	2.8

1 Diets: CD = control diet 0% microalgae; CD3 = control diet + 3% microalgae; RS3 = exchange of soybean and sunflower by rapeseed + 3% microalgae.

2 Provided per kilogram of complete diet: vitamin A 10,000 IE; vitamin D_3_ 2,000 IE; vitamin E 25 mg; vitamin K_3_ 1.5 mg; vitamin B_1_ 1.0 mg; vitamin B_2_ 3.5 mg; vitamin B_6_ 1.0 mg; vitamin B_12_ 15 µg; niacin 30 mg; D-pantothenic acid 12 mg; choline chloride 350 mg; folic acid 0.8 mg; biotin 0.1 mg; iron 50 mg; copper 10 mg; manganese 60 mg; zinc 54 mg; iodine 0.7 mg; selenium 0.1 mg.

3 CVB matrix values (CVB, 2018) were used for diet formulations.

### Measurements

All pens were group weighed at the beginning and end of the experimental period (d 0 and 28). On a weekly basis, the feed intake, laying percentage, and egg weight were determined. Feed conversion ratio was calculated on basis of the feed intake and egg weight. From d 22 to d 27 all eggs per pen were collected. Per collection d, 5 random eggs were collected and pooled per 3 d (d 22, 23, 24, and d 25, 26, 27). Total egg weight, scale weight, albumen weight (fresh and dry), and yolk weight (fresh and dry) were determined. The pooled yolk was dried to obtain the dry matter content. An external laboratory determined the fatty acid composition in the yolk (NutriControl BV, Veghel, the Netherlands). Litter material was removed at d 23 and excreta were collected from d 24 to d 27. Representative samples were taken of all five provided diets. Feed was dried and excreta were freeze dried and analyzed for dry matter content, crude ash, crude protein, crude fat, crude fiber, fatty acid composition, and titanium by an external laboratory (NutriControl BV). At d 28 two laying hens per pen were sacrificed and 1 cm of tissue from the middle of the intestine part, both from the jejunum and colon, were collected. Samples were analyzed for villi length and crypt depth by an external laboratory (Gezondheidsdienst voor Dieren, Deventer, the Netherlands). Furthermore, blood samples were taken from these 2 laying hens to obtain blood plasma for IL-13 and haptoglobin determination.

### Chemical Analyses

Chemical analyses were performed by an external laboratory (NutriControl). For determination of the DM content in digesta, samples were freeze-dried according to International Organization for Standardization (**ISO**) method number 6496 (1998). Following freeze-drying, samples were ground to pass a 1 mm screen and kept for analysis. Air-dry samples were dried in a forced air oven at 103°C to a constant weight according to ISO 6496 (1998). Kjeldahl nitrogen content in feed was measured according to ISO 5983 (1997) in fresh samples. CP content was calculated as nitrogen × 6.25. For determining crude ash content, feed samples were incinerated at 550°C in a muffle furnace according to ISO 5984 (2002a,b,c). Crude fiber was measured according to an internal protocol based on regulation (EC) No. 152/2009, Appendix 3, Method I. Titanium oxide was determined according to the method developed by Short et al. (1996) and further refined by Myers et al. (2004). This method is based on digestion of the sample in sulphuric acid and addition of hydrogen peroxide to produce an intense orange/yellow color that is read calorimetrically at 408 nm by use of an UV- visible spectrophotometer (Varian, CARY 50 probe). Furthermore, the fatty acid composition of the microalgae, diets and excreta were determined according to ISO/TS 17764-1/2 (2002a,b). The fatty acids composition was analyzed in dry egg yolk of eggs collected in the last experimental week (wk 4). The fatty acid composition is presented on basis of 100 g dry egg yolk as well as in mg per egg.

### Calculations

The rate of lay was calculated by dividing the total number of eggs in 1 wk by the production days (number of birds multiplied by the number of days when eggs were produced). Feed conversion ratio was calculated by dividing the total feed intake by the total egg mass of one pen. Based on the analyzed content in the experimental diets and excreta, the total tract nutrient retention of DM, ash, organic matter (=DM – ash), crude fat, and crude fiber were calculated using the following equation:

$$\text{Nutrient}\;\text{retention}\;\left(\%\right)=100-\left[100\times \left(M_{\text{diet}}\times \text{Nutrien}t_{\text{excreta}}\right)/\left(M_{\text{excreta}}\times \text{Nutrien}t_{\text{diet}}\right)\right]$$

with M_diet_ and M_excreta_ as the analysed concentrations of marker (TiO_2_) in the diet and excreta (g/kg DM) and Nutrient_diet_ and Nutrient_excreta_ are the analyzed concentrations of nutrient in the diet and excreta (g/kg DM).

### Statistical Analysis

The data were analysed with analysis of variance (**ANOVA**) as a randomized block design using GenStat statistical software. All variables and covariables were expressed as average of the pen. For all variables this average consisted of 8 laying hens (the complete pen), except for the blood and gut tissue related parameters, of which the average consisted of 2 laying hens. The general model, as depicted below, includes inclusion of the microalgae in the diet as fixed effects and room and block (place within the room) as random effects:

$$Y_{\text{ijk}}=\mu +\text{Roo}m_{i}+\text{Bloc}k_{j}+\text{Die}t_{k}+e_{\text{ijk}}$$

in which:

Y_ijkl_= dependent variable,
µ= overall mean
Room_i_ = room effect (j=1,2)
Block_j_ = block effect (k=1, 2, 3, 4, 5, 6)
Diet_k_  = effect of dietary treatment, (l= 1, 2, 3, 4, 5)
e_ijk_  = residual error.

This model was used to analyze the results of each of the performance parameters (body weight, feed intake, rate of lay, egg weight, and feed conversion ratio), nutrient retention, egg characteristics (shell, yolk, and albumin weights and ratio), and fatty acid compositions. Parameters were tested for normal distribution before analyses. The pen was the experimental unit for the response parameters. For the performance parameters, nutrient retention, egg characteristics and fatty acid composition, analyses were performed to determine linear and quadratic effects of dietary inclusion of the microalgae (dose-response). A Fisher unprotected *t*-test has been used for comparison of treatment means.

Because of the two objectives in this study, treatments B and C were not comparable with treatment E. Due to the influence treatment E had on the averages, variances and analysis, treatment E was excluded from the analyses of performance egg characteristics and fatty acid composition.

Intestinal morphometry and systemic inflammatory markers were only analysed comparing treatments A, D and E. The statistics of the measured systemic inflammatory blood concentration levels of IL13 and haptoglobin and the intestinal morphometry were analysed in GraphPad Prism (v8.2.1; GraphPad Software, San Diego, CA). Normality tests that is, Shapiro-Wilk and Kolmogorov-Smirnov test were carried out in the blood concentration levels of IL13, haptoglobin and the intestinal morphometry in each treatment. To find the significant differences, we separately compared treatment D and E with the reference diet (treatment A). When both the treatments passed the normality test, parametric *t*-test was performed. When the compared treatments failed the normality test then nonparametric that is, Mann-Whitney test were performed. *P* value < 0.05 was considered significant.

## RESULTS

The experiment was conducted according to protocol without any deviations. The laying hens arrived healthy and were distributed with a maximum of 5% average group body weight deviation between pens. Total mean body weight of the hens in one pen was 9,327 g (average of 1,166 g per hen). Due to transport, most hens stopped laying for a few days which resulted in a very low laying percentage in week one (27.3%). After this one week of adaptation, laying performance returned to normal according to the management guide (H&N International GmbH, 2018). The first week was therefore excluded from analyses. Laying percentage in week 2, 3 and 4 (91.0, 94.0, and 96.0% respectively) did not differ from H&N Super Nick performance objectives. During the experiment, no veterinary treatments have been executed and mortality was very low (0.42%, one bird was found dead).

### Content of the Diets

The calculated and analyzed contents of the experimental diets are presented in Table 3. The analysed values for DM, crude protein, crude ash, and starch were close to the calculated values of the diets. The analysed crude fat content of the A diet was close to the calculated value, however, the analyzed crude fat content for diets B, C, D, and E was respectively 4, 5, 5, and 5 g/kg lower than calculated. Crude fiber content of the D and E diets were close to the calculated values, whereas the analyzed values for A, B and C were 3 g/kg, 2 g/kg, and 2 g/kg higher than the calculated value, respectively. The analyzed values for sugar were higher in all 5 diets. Furthermore, the titanium content of the diets was 0.4, 0.3, 0.3, 0.3, and 0.2 g/kg lower than the calculated content of A, B, C, D, and E, respectively. The DM, crude protein, crude ash, and crude fiber content of the excreta did not differ amongst treatments (data not shown). However, the excreta of the birds that were fed the rapeseed + 3% microalgae diet (treatment E) had a higher crude fat content compared to the other treatments (*P* = 0.048). Furthermore, the titanium content of the excreta from birds fed treatment E differed from birds fed the control diet, and the diet containing 1% microalgae (*P* = 0.043).

**Table 3:** Calculated (Calc) and analyzed (Ana) nutrient content of experimental diets (g/kg).

	A		B		C		D		E	
Diet^1^	Calc	Ana	Calc	Ana	Calc	Ana	Calc	Ana	Calc	Ana
Dry matter	891	900	891	902	892	904	891	902	892	904
Crude protein	161	157	161	162	161	159	161	162	161	159
Crude ash	128	133	127	127	127	131	127	127	127	131
Crude fat	58	56	56	50.5	69	64	56	51	69	64
Crude fiber	37	40	36	38	43	44	36	38	43	44
Starch	379	384	379	394	354	362	379	394	354	362
Sugar	30	34	29	32	33	37	29	32	33	37
TiO_2_	5	4.6	5	4.7	5	4.8	5	5	5	5

1 Diets: CD = control diet 0% microalgae; CD3 = control diet + 3% microalgae; RS3 = exchange of soybean and sunflower by rapeseed + 3% microalgae.

### Fatty Acid Composition in Microalgae and Dietary Treatments

The levels of DHA, EPA and major fatty acids groups of the dried microalgae and the treatments are presented in Table 4, the full fatty acid composition of the microalgae can be found in Appendix 1. The means of treatment E can be found in Appendix 2. In neither the dry material of the microalgae, nor the dietary treatments DHA was measured. The EPA level in the diets increased in accordance with the supplementation of the microalgae, thus the omega 3 level increased accordingly. However, the values of EPA in the diets were lower than expected on basis of its concentration in the microalgae which may be due to processing and storage of the diets.

**Table 4:** Fatty acid composition (g/kg) of dry *N. limnetica* biomass and the dietary treatments.

Treatment^1^	*N. limnetica*	A	B	C	D	E
DHA	0	0	0	0	0	0
EPA	35.8	0	0.28	0.47	0.71	0.7
Monounsaturated	64.9	17.9	17.9	16	15.7	21.3
Poly unsaturated	54	14.3	15	13.9	14.3	15.9
Saturated	38.8	20.2	19.7	17	16.1	21.6
Omega 3	36.2	0.67	0.95	1.04	1.26	1.54
Omega 6	16.7	13.6	14	12.8	12.9	14.2
Omega 9	21.7	17.3	17.1	15	14.3	19.3

1 Treatment: A = control diet + 0% microalgae; B = control diet + 1% microalgae; C = control diet + 2% microalgae; D = control diet + 3% microalgae; E = exchange of soybean and sunflower by rapeseed + 3% microalgae.

**Appendix 1:** Full fatty acid composition (g/kg) of dry biomass *N. Limnetica* used in this study

Fatty Acid	Result	Fatty Acid	Result
anteiso-C15:0	0.04	C20:4n6	9.13
anteiso-C16:0	0.04	C20:5n3 EPA	35.83
anteiso-C17:0	0.03	C21:0	0.00
C10:0	0.15	C22:0	0.07
C10:1	0.00	C22:1n11	0.00
C11:0	0.01	C22:1n9	0.00
C12:0	0.30	C22:2n6	0.00
C12:1	0.00	C22:3n3	0.04
C14:0	4.75	C22:4n6	0.00
C14:1n5	0.07	C22:5n3	0.05
C14:1n9	0.11	C22:5n6	0.04
C15:0	0.79	C22:6n3 DHA	0.04
C15:1	0.05	C23:0	0.10
C16:0	30.91	C24:0	0.01
C16:1n7	41.45	C24:1n9	0.00
C16:1n9	0.10	C4:0	0.00
C16:3	0.00	C5:0	0.00
C16:4	0.12	C6:0	0.04
C17:0	0.31	C7:0	0.00
C17:1	0.75	C8:0	0.26
C18:0	0.39	C9:0	0.05
C18:1 trans	0.11	CLA 10trans 12cis	0.17
C18:1n	0.73	CLA 9cis 11trans	0.00
C18:1n9	21.51	elutable	157.71
C18:2 trans	0.96	Monounsaturated	64.87
C18:2n6	5.98	Iso C14:0	0.07
C18:3n3	0.00	iso-C15:0	0.09
C18:3n6	0.87	iso-C16:0	0.00
C18:4n3	0.04	iso-C17:0	0.03
C19:0	0.02	iso-C18:0	0.30
C20:0	0.08	Polyunsaturated	54.04
C20:1n11	0.00	Omega 3	36.22
C20:1n9	0.00	Omega 6	16.70
C20:2n6	0.12	Omega 9	21.72
C20:3n3	0.00	Trans	1.24
C20:3n6	0.57	Saturated	38.80
C20:4n3	0.11

**Appendix 2:** Means of production performance, egg characteristics and fatty acid composition pf treatment E; basal diet with exchange of soybean and sunflower by rapeseed + 3% microalgae

		Treatment E
Average body weight per hen (g)		
	Start	1161
	End (28 days)	1463
	Growth	302
Feed intake (g/hen/day)		
	wk 1	104
	wk 2	130
	wk 3	125
	wk 4	131
	average^1^	128
Rate of lay (%)		
	wk 1	23.2
	wk 2	88.7
	wk 3	92.6
	wk 4	95.8
	average^1^	92.4
Average egg weight per egg (g)		
	wk 1	48.1
	wk 2	54.0
	wk 3	57.3
	wk 4	57.0
	average^1^	56.1
Feed conversion ratio (%)		
	wk 1	9.9
	wk 2	2.7
	wk 3	2.4
	wk 4	2.8
	average^1^	2.6
Fresh weights (g per egg)		
	Shell	7.4
	Yolk	13.9
	Albumin	35
Ratio (%)		
	Shell	13
	Yolk	24.8
	Albumin	62.4
Monounsaturated		482.1
Polyunsaturated		157.2
Saturated		355.9
Omega 3		20.6
Omega 6		134.5
Omega 9		415.9
EPA (mg in the egg)		5.3
DHA (mg in the egg)		56.7

1 Due to severe drop in production, wk 1 is excluded from the average

### Total Tract Nutrient Retention of the Diets

The inclusion of microalgae did not affect total tract nutrient retention of the diets. Inclusion of rapeseed tended to decrease the crude fat retention compared to all other treatments (*P* = 0.063) and numerically decreased all retention coefficients (Table 5).

**Table 5:** Total tract nutrient retention (%) of diets with increasing inclusions (0, 1, 2, 3%) of microalgae.

Treatment^1^	A	B	C	D	E	SEM^2^	*P value*^3^
Dry matter	69.1	67.2	70.6	70.1	60.6	4.3	0.16
Crude protein	50.3	46.4	52.5	51.5	40.7	6.9	0.44
Crude ash	46.9	39	48.7	45.3	31.2	7.8	0.2
Crude fat	71.8	69.2	68.7	67.9	57.2	4.9	0.063
Crude fiber	7.3	−9.7	8.2	8.1	−14	13.7	0.31

1 Treatment: A = control diet + 0% microalgae; B = control diet + 1% microalgae; C = control diet + 2% microalgae; D = control diet + 3% microalgae; E = exchange of soybean and sunflower by rapeseed + 3% microalgae.

2 SEM = average standard error of the mean.

3 Pairwise differences are marked with superscripted indices when significant differences (*P* < 0.05) were observed.

### Laying Hen Performance

Because of the different objectives (effect of microalgae on performance vs. influence on intestinal morphometry and systemic inflammatory markers) and dietary differences, treatment B, C should not be compared with treatment E. Due to the influence treatment E has on the averages, variances and analysis, treatment E was excluded from the analyses of performance on egg characteristics and fatty acid composition. Table 6 represents the effects of the inclusion of microalgae on laying hen performance. The average increase in body weight of the individual hens, as well as the feed conversion ratio and the average egg weight were not affected by inclusion of the microalgae in the diet. The average feed intake per hen per day, however, was affected by the dietary treatments. Inclusion of 2% and 3% microalgae resulted in a higher feed intake compared to the control group (*P* = 0.001). The rate of lay of birds fed 2 or 3% microalgae was respectively 5.8 and 4.4% (*P* = 0.018) higher compared to the control group. In the first 3 wk, the rate of lay tended to normalize faster upon inclusion of the microalgae in the diet which might suggest a faster recovery from stress conditions during transport (*P* = 0.092). Although not significant, the numerical difference in feed intake between wk 1 and wk 2 correspond to a faster recovery due to the supplementation of the microalgae. The means of treatment E can be found in Appendix 2.

**Table 6:** Effects of increasing inclusions (0, 1, 2, 3%) of microalgae on laying hen performance.

Treatment^1^	A (0%)	B (1%)	C (2%)	D (3%)	Average treatments	SEM^2^	*P-value*^4^				
							Treatment				
								Linear	Quadratic	Week	Week × Treatment
Average body weight per hen (g)											
Start	1,175	1,162	1,161	1,171		16.8	0.8	0.82	0.34		
End (28 d)	1,495	1,464	1,483	1,487		18.8	0.42	0.92	0.2		
Growth	321	302	322	316		33.3	0.7	0.92	0.64		
Feed intake (g/hen/day)											
wk 1	106	106	109	106	107						
wk 2	119^B^	126^A^	125^A^	126^A^	124	2	0.007	0.004	0.034		
wk 3	117^B^	121^AB^	126^A^	124^A^	122	2.7	0.051	0.019	0.16		
wk 4	122	120	126	124	123	2.3	0.06	0.043	0.96		
Average^3^	119^C^	122^BC^	126^A^	125^AB^		1.6	0.004	0.001	0.08	0.1	0.24
Rate of lay (%)											
wk 1	31.3	24.4	30.4	27.1	28.3						
wk 2	86.6	89.6	97.3	92.9	91.6^b^	4.32	0.12	0.07	0.24		
wk 3	90.5	96.7	97	93.2	94.3^ab^	2.62	0.07	0.33	0.016		
wk 4	94.8	95.8	94.8	98.9	96.1^a^	2.23	0.24	0.13	0.34		
Average^3^	90.6^B^	94.1^AB^	96.4^A^	95.0^A^		1.83	0.039	0.018	0.08	0.013	0.09
Average egg weight per egg (g)											
wk 1	47.6	48.0	48.4	46.9	47.7						
wk 2	54.2	54.7	54.2	53.7	54.2	0.74	0.61	0.42	0.35		
wk 3	57.8	57.7	57.5	56.8	57.4	0.77	0.58	0.22	0.56		
wk 4	57.3	57.1	57.5	56.4	57.1	0.79	0.54	0.36	0.48		
Average^3^	56.4	56.5	56.4	55.6		0.65	0.53	0.24	0.39	<0.001	0.77
Feed conversion ratio (%)											
wk 1	7.8	10.3	8.1	8.7	8.7						
wk 2	2.6	2.6	2.4	2.5	2.52^c^	0.1	0.570	0.560	0.580		
wk 3	2.3	2.2	2.2	2.3	2.25^b^	0.1	0.260	0.260	0.110		
wk 4	2.2	2.2	2.3	2.2	2.61^a^	0.1	0.370	0.530	0.700		
Average^3^	2.5	2.4	2.4	2.5		0.06	0.750	0.710	0.310	<0.001	0.21

1 Treatments: A = control diet 0% microalgae; B = control diet + 1% microalgae; C = control diet + 2% microalgae; D = control diet + 3% microalgae;

2 SEM = average standard error of the mean. Wk 1 is excluded from the average.

3 Due to severe drop in production, wk 1 is excluded from the average and analysis.

4 Pairwise differences are marked with superscripted indices when significant differences (*P* < 0.05) were observed.

AB Means within a row with no common superscript differ (*P* < 0.05).

ab Means within a column with no common superscript differ (*P* < 0.05).

### Egg Characteristics

**Figure 1:**
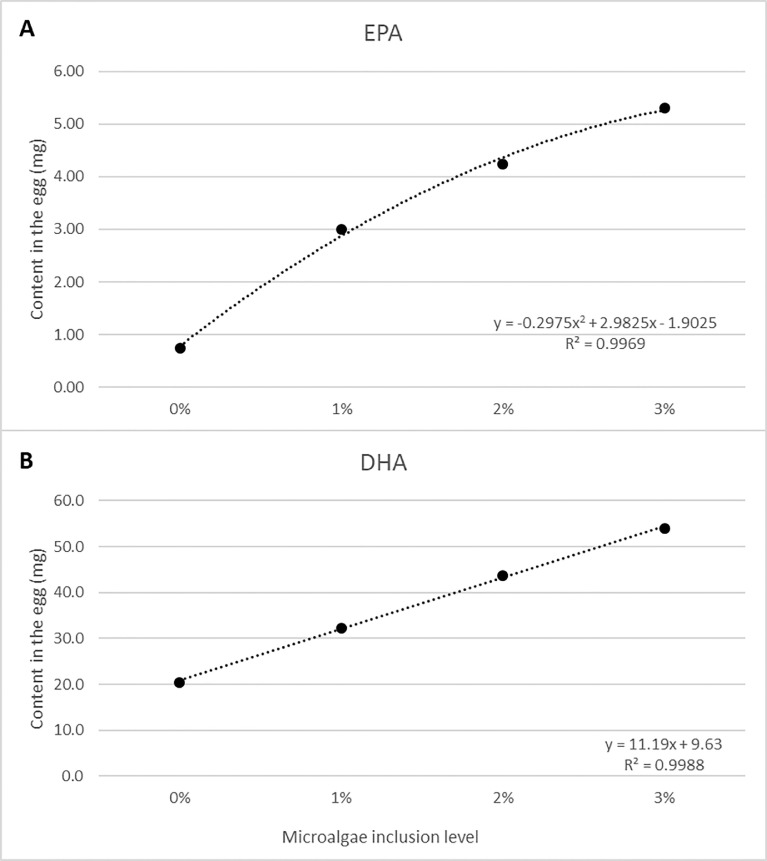
Relation between microalgae inclusion level in the feed and the EPA (A) and DHA (B) content in the egg.

Inclusion level of microalgae had neither effect on the weight of the shell, yolk or albumen nor on the ratio of these parts of the egg (Table 7). The EPA content of the eggs was relatively small and was both linear and quadratically affected by the increasing inclusion of microalgae (*P* < 0.00; Figure 1A). The DHA content was only linear affected by the increasing inclusion of the microalgae (*P* < 0.001; Figure 1B).

**Table 7:** Effects of increasing inclusions (0, 1, 2, 3%) of microalgae on egg characteristics

							*P-value*	
Treatment^1^		A (0%)	B (1%)	C (2%)	D (3%)	SEM^2^	Linear	Quadratic
Fresh weights (g per egg)								
Shell		7.7	7.6	7.8	7.6	0.1	0.3	0.55
Yolk		15.1	14.6	15	14.6	0.3	0.16	0.84
Albumin		34.4	34.4	34.5	34.1	0.7	0.76	0.58
Ratio (%)								
Shell		13.4	13.4	13.5	13.3	0.1	0.57	0.85
Yolk		26.5	25.9	26.3	26.1	0.3	0.46	0.38
Albumin		60.3	61	60.5	60.8	0.4	0.42	0.51

1 Treatment: A = control diet 0% microalgae; B = control diet + 1% microalgae; C = control diet + 2% microalgae; D = control diet + 3% microalgae.

2 SEM = average standard error of the mean.

### Fatty Acid Composition of the Yolk

The effects of the experimental diets on the monounsaturated, polyunsaturated, saturated, omega 3, omega 6 and omega 9 fatty acids of the yolk are presented in Table 8. The different dietary treatments did not affect the monounsaturated fatty acids, polyunsaturated fatty acids and omega 9 fatty acids. The omega-3 fatty acid content, however, was both linearly and quadratically affected by the dietary treatments (*P* < 0.001). With an increase of algal biomass in the feed, the omega-3 content in the yolk increased as well. All the treatments differed from each other. Our results show that the plateau of the effects of including these microalgae on the omega-3 content of the yolk is higher than 3%. The omega 6 content of the yolk was negatively affected by the 2 and 3% inclusion of microalgae (*P* = 0.004). The saturated fatty acid content was approximately 230 mg higher when 2% and 3% microalgae were included, compared to no supplementation of microalgae (*P* = 0.022).

**Table 8:** Effects of increasing inclusions (0, 1, 2, 3%) of microalgae on yolk fatty acid composition (g/100 g dry yolk).

Treatment^1^						*P*-value^3^	
Fatty acids	A	B	C	D	SEM^2^	Linear	Quadratic
Monounsaturated	26.5	26.64	26.49	26.78	0.174	0.22	0.57
Polyunsaturated	8.11	8.27	8.11	8.26	0.174	0.61	0.97
Saturated	20.17^b^	20.35^ab^	20.42^a^	20.39^a^	0.09	0.022	0.126
Omega 3	0.43^d^	0.67^c^	0.87^b^	1.06^a^	0.014	<0.001	0.022
Omega 6	7.56^a^	7.47^ab^	7.13^bc^	7.09^c^	0.166	0.004	0.84
Omega 9	22.9	22.8	22.77	22.66	0.198	0.6	0.95

1 Treatment: A = control diet 0% microalgae; B = control diet + 1% microalgae; C = control diet + 2% microalgae; D = control diet + 3% microalgae.

2 SEM = average standard error of the mean.

3 Pairwise differences are marked with superscripted indices when significant differences (*P* < 0.05) were observed.

abcd Means within a row with no common superscript differ (*P* < 0.05).

### Intestinal Morphometry and Systemic Inflammatory Markers

**Figure 2:**
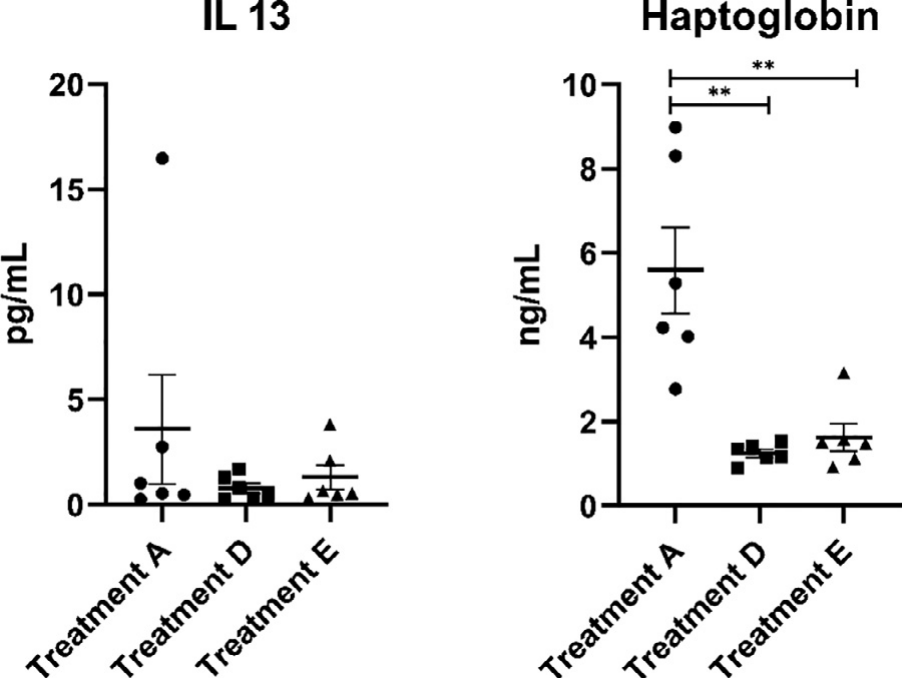
Boxplot based on the effects of 3% microalgae inclusion (treatment D) and 3% microalgae inclusion + mild dietary rapeseed + sunflower challenge (treatment E) compared to the reference diet (treatment A) on systemic inflammatory parameters. Each dot, square and triangle represents the average per pen (two birds per replicate). ** represent a significance of *P* < 0.01 between the treatments indicated by the horizontal lines.

**Figure 3:**
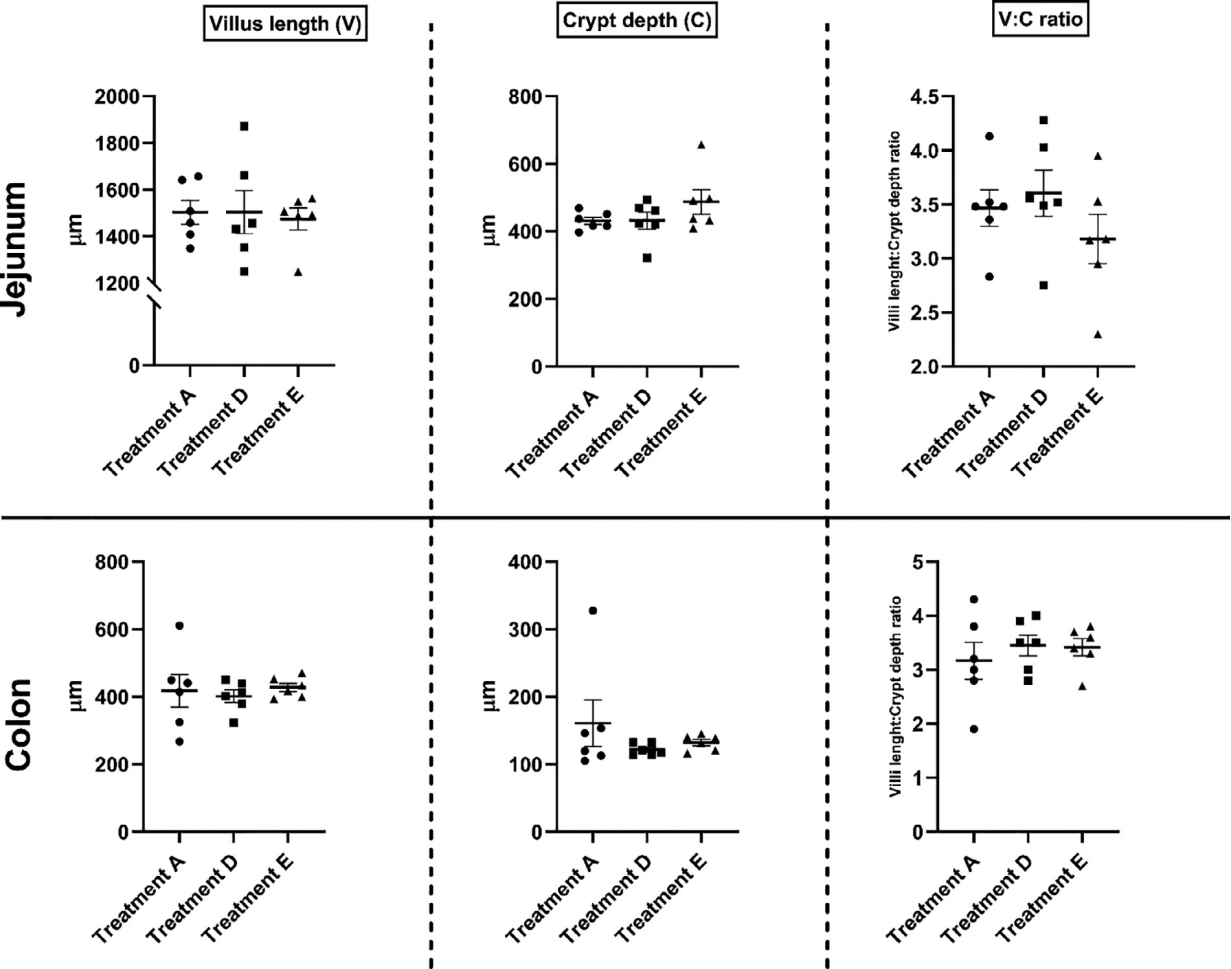
Boxplot based on the effects of 3% microalgae inclusion (treatment D) and 3% microalgae inclusion + mild dietary rapeseed + sunflower challenge (treatment E) compared to the reference diet (treatment A) on gut morphometry in the jejunum and colon. Each dot, square and triangle represents the average per pen (2 birds per replicate).

To investigate whether the inclusion of 3% microalgae in the diet of laying hens leads to a change in systemic immunity, IL13, and haptoglobin were measured in the blood plasma of hens fed diets D and E and compared with the values measured in hens fed the reference diet A (Figure 2). Blood plasma concentration of haptoglobin was low (*P* < 0.05) in both groups supplemented with microalgae that is, treatment D and E, compared to treatment A. A notable finding was that hens which received the rapeseed meal as a mild dietary challenge in addition to the 3% microalgae supplement (i.e., diet E) had no difference (*P* > 0.05) in blood plasma concentration of haptoglobin compared to hens that received 3% microalgae (i.e., diet D). No difference was observed in blood plasma concentrations of IL13. Intestinal morphometry of both the jejunum and colon was not affected by dietary challenge or inclusion of 3% microalgae in the laying hen diet (Figure 3).

## DISCUSSION

### Diets and Retention

The diets for this study were produced in agreement with the calculated nutrient content. The nutrient content of the five diets differed minimally between each other, except where the difference where due to the inclusion of the microalgae. The nutrient retention of the diets was not affected by the inclusion of *N. limnetica*. Only the retention of fat tended to be lower in treatment E, indicating that rapeseed meal negatively affects the fat retention, which cannot be counteracted for by the inclusion of 3% microalgae. Overall, the nutrient retention found in this experiment was considered normal. The nutrient retention of crude fibre is very variable and can result in negative values. Studies researching the retention of other microalgae in chickens found contradictory effects. Choi et al. (2017) and Abdelnour et al. (2019) supplemented broiler diets with *Chlorella vulgaris* and found a higher energy digestibility, but no differences in dry matter digestibility. On the other hand, Park et al. (2018) found a linear increase in apparent total tract digestibility of dry matter and nitrogen of broilers fed with diets with increasing inclusion of *Arthrospira platensis* (Spirulina). Similar results were found in weaned piglets, fed with *Schizochytrium spp.* Supplementation (Kibria and Kim, 2019) and piglets fed with Spirulina or *C. vulgaris* (Furbeyre et al., 2017). Furbeyre et al. (2017) also found an increase in total tract digestibility for gross energy. In vitro work on several microalgae (Spirulina, C*. vulgaris, Tetraselmis suecia*, and *Phaeodactylum tricornutum*) did not show any differences in digestibility (Batista et al., 2017).

### Production Performance

As different microalgae have their own structure, components and function, the overall outcomes of this and other studies are not conclusive for microalgae in general. In our study, the rate of lay was higher when *N. limnetica* was provided. Most other studies found either no effects, or also an improvement on the egg production (Park et al., 2015; Choi et al., 2018). However, none of the above studies, nor our study was designed to determine the mode of action to explain an improvement of egg production. It might be speculated that the essential amino acid content or other health beneficial microalgal compounds have a positive influence on production. Another possibility is the influence of the microalgae on the microbiome (Janczyk et al., 2009). The microbiome composition and the metabolites provided by the microbiome could influence the egg production process.

Inclusion of microalgae in the diet did not affect the body weight of the laying hens. This is in line with most of the results found in other studies (Bruneel et al., 2013; Ao et al., 2015; Ekmay et al., 2015; Neijat et al., 2016; Moran et al., 2019). Only Lemahieu et al. (2013) found a 5% decrease in body weight when feeding 2 levels of *N. Oculate* between 2.5 and 8.6% supplementation. However, no explanation was given for this decrease. In our work, the feed intake was higher in the treatments with 2 and 3% supplementation of *N. limnetica*. This is in contrast with other studies, where either no effect or a decrease in feed intake has been found (Halle et al., 2009; Ao et al., 2015; Ekmay et al., 2015; Neijat et al., 2016). Compared to the reference diet the feed intake in this study, increased with 7 or 6 g per hen per day when hens were fed the C or D diets, respectively. Laying hens usually have a lower feed intake at the beginning of the production period, which is lower than required for maintenance and production (Leeson and Summers, 2005). Thus, a voluntary increased feed intake is positive for the short-term (increase of production) and the long-term (persistency). In this study neither effects of the microalgae on the feed conversion ratio nor on the average egg weight were measured. Similar results were reported in other studies (Halle et al., 2009; Ao et al., 2015; Neijat et al., 2016; Wu et al., 2019).

### Egg Characteristics

In this study, no effects were found of the inclusion of microalgae on the shell, yolk, or albumin weights or the ratio of these egg components. This is in line with other egg characteristics studies which also found no differences (Fredriksson et al., 2006; Lemahieu et al., 2013; Ao et al., 2015; Ekmay et al., 2015; Neijat et al., 2016; Wu et al., 2019). This study did not measure any olfactory characteristics of the eggs; no systematically observed values can be reported. However, in the eggs that were consumed, a change in color of the yolk was noted upon inclusion of the microalgae. Some of the yolks were greenish or had green hints. In this work we did not study which level of *N. limnetica* inclusion and which specific compound caused the yolk colouring. Consequence of this possible colouring may be that, without proper information, egg consumers will be hesitant to buy and consume such green eggs. It could be possible to use other natural colourings in the diet (i.e., carotene) to counteract the green colouring. Other studies also found differences in yolk color when providing microalgae (Ginzberg et al., 2000; Halle et al., 2009; Zheng et al., 2012; Bruneel et al., 2013; Wu et al., 2019).

### Fatty Acid Composition of the Eggs

This study showed clear effects of the inclusion of *N. limnetica* on the EPA and in particular the DHA content in the yolk. Consequently, the total omega 3 content of the egg is affected by the microalgae inclusion levels as well. Results reported by Nitsan et al. (1999) showed that addition of 1.0% *Nannochloropsis* spp. To the diet elevated the DHA content of the eggs by almost 25%. Wu et al. (2019) also studied *Nannochloropsis spp*. Supplementation (1, 2, 4, and 8%) in laying hens’ diets. Similar to the results of the current study, they also reported increasing DHA and EPA content while increasing the supplementation in the diet. Increasing levels of DHA levels are also found in the study of Ao et al. (2015) upon supplementing increasing levels of commercial microalgae. Similar results were found by Lemahieu et al. (2013), who tested 4 microalgae (*Phaeodactylum tricornutum, Nannochloropsis oculata, Isochrysis galbana, Chlorella fudca*) with 2 inclusion levels (0.125 and 0.250%). Lemahieu et al. (2013) also reported DPA in all microalgae enriched eggs. DPA is an intermediate in the conversion process of EPA to DHA. According to Nitsan et al. (1999) and Lemahieu et al. (2013), it seems that, due to the much higher amounts of DHA compared to EPA found in the eggs, microalgal omega 3 fatty acids are first converted to DHA, before those fatty acids are deposited in the egg yolk. This hypotheses and results are confirmed by Bruneel et al. (2013), who also found low levels of EPA and high levels of DHA. The results of the current study contribute to this hypothesis as well, since also in this study the DHA levels were much higher than the EPA levels. However, the levels in eggs found in this study (DHA: 20.5–53.9 mg/egg and EPA: 0.7–5.3 mg/egg), are a bit lower, but comparable to levels found by Lemahieu et al. (2013) (approx. DHA: 48–67 mg/egg and approx. EPA: 3.1–6.2 mg/egg). On basis of dry yolk our results are considerably higher than the levels found in the studies by Wu et al. (2019) (average DHA: 42.0–111.6 mg/g yolk and average EPA: 7.75–14.92 mg/yolk). As Figure 1 shows, the DHA and EPA content did not plateau yet upon increases algae content. Thus, the optimum of *N. limnetica* inclusion in laying hen diets to increase the DHA and EPA content in the yolk is considerably higher than 3%, based on the amount of algal omega-3 fatty acids included in the feed. Wu et al. (2019) supplemented the diets with 1, 2, 4, and 8% of *Nannochloropsis* spp., and saw the DHA content plateau in time for each group but on different levels, dependent on the initial level that was provided in the diet. The study of Wu et al. (2019) did not report quadratic testing of the doses. However, the results show that inclusion of 4 and 8% is possible and indicate that higher levels of microalgae result in similar dynamics, but at a different level. Nevertheless, the effects on performance are not properly researched when including even higher amounts of microalgae, thus these possible consequences should be studied before using high inclusions of microalgae in practice. Furthermore, high inclusion is also dependent on the amount and bioavailability of omega 3 fatty acids in the microalgae. Based on the average intake of feed and the number of eggs per timeperiod, the estimated efficiency of algal EPA + DHA deposition in the eggs was about 35% for all algal inclusion levels (data not shown). This value indicates a quite well bioavailability of the algal biomass which was only dried before inclusion in the feed and is higher than the efficiency reported by Lemahieu et al. (2013), who found a 20% efficiency of *N. Oculata*.

### Intestinal Histomorphometry and Systemic Inflammatory Markers

In this study, the histomorphometric characteristics of the intestine and the changes in the concentrations of APP (haptoglobin) and cytokines (interferon-γ; data not shown because the measured concentrations were below the detection limit; and IL-13) in the blood of laying hens fed the experimental diets were recorded and compared with the group fed the reference diets to look for any adverse effects of the experimental diets. Rapeseed was used as a mild nutritional challenge for the gastrointestinal tract. Due to the complex fibre matrix and heat treatment at production of the diets, the digestibility of diets with high levels of rapeseed meal is lower. More distally in the gut, delayed digestion of nutrients results in more substrate arriving in the large intestine, causing more microbial fermentation. Extensive fermentation is undesirable and a risk for gut health. Neither the rapeseed challenge, nor the microalgae affected the morphology of the jejunum or colon. Other studies found improvements in gut morphology when microalgae were incorporated into chicken feed. Broilers fed a basal diet enriched with Chlorella by-products at 1% (Mirzaie et al., 2020) or 2.5, 5, and 7.5% (Kang et al., 2017) were found to have greater jejunum villi height and crypt depth. However, Mirzaie and colleagues observed no significant difference in the intestinal histomorphometry in birds fed diets containing 2% Chlorella by-products (Mirzaie et al., 2020). Combining the results of this study with those of previous studies, it appears that the effects of microalgae on intestinal histomorphometry depend on the type and content of microalgae. We did not measure concentrations of the cytokine IFN-γ above the limit of detection in the blood of birds receiving treatment A, D, or E. No significant differences were observed between treatments D and E and the reference diet A regarding IL -13. This observation of cytokine and chemokine levels confirms that the dietary treatments did not promote cell-mediated immune responses or induce physiological changes due to inflammation in tissues. Furthermore, we did not observe any signs of inflammation or pathophysiological events in histomorphometry, which is consistent with the observations for cytokines and chemokines. In addition, we observed significantly lower haptoglobin concentration in treatment groups D and E compared to the reference diet, that is, treatment A. Although intersample variability was high in treatment A, the results suggest that treatment D and E resulted in a different inflammatory response compared to treatment A. Higher haptoglobin concentrations reflect inflammatory or infection status due to microbes (pathogenic bacterial, protozoal or viral loads) in broiler chickens (Garcia et al., 2009; Georgieva et al., 2010; Asasi et al., 2013). However, quails infected with fungal infection (aspergillosis) were found to have lower haptoglobin levels compared to the uninfected groups (Goetting et al., 2013). This suggests that in birds, discrepancies in the direction of haptoglobin change may depend on the type of infection. Although the direction of haptoglobin changed in this study is consistent with that of a study of fungal infections in quail, we did not detect high mortality or other signs indicating a negative impact on the health or productive performance of laying hens in our study. Overall, the histomorphometry, IL13, and haptoglobin results indicate that the experimental diet most likely does not contain any hazardous substances that could affect intestinal tissues or inflammatory markers to the extent that the health or performance of the laying hens would be affected during the 4-wk experimental period.

## CONCLUSIONS

From this study it can be concluded that increasing inclusion of *N. limnetica* biomass in laying hen diets increases the DHA and EPA levels of the yolk and has small effect on the hens’ performance. The rate of lay was higher when 2 or 3% *N. limnetica* was provided and the feed intake was increased in the treatments with 2 and 3% supplementation of the microalgae. This was possibly caused by the lower crude fat content of the algae diets. Inclusion of 3% microalgae resulted in lower haptoglobin levels in both the control and rapeseed diets. Other performance parameters, the intestinal morphometry and also the diet digestibility was not affected by the inclusion of the microalgae, indicating these microalgae could replace some protein sources without negative effects. Although there are no official guidelines for the intake of long-chain omega-3 fatty acids for humans yet, a minimum of 160 to 250 mg and a maximum of 3,000 mg of combined EPA and DHA per day is indicated for human consumption. Consumption of omega-3 enriched eggs with these microalgae can contribute to such an intake. In this study, we did not reach the optimal inclusion level of *N. limnetica* in laying hen diets to affect the omega 3 fatty acids in the yolk. However, the literature available on these topics is scarce, thus further research confirming these results are necessary.
